# A qualitative exploration of autistic mothers’ experiences I: Pregnancy experiences

**DOI:** 10.1177/13623613221132435

**Published:** 2022-11-03

**Authors:** Sarah Hampton, Joyce Man, Carrie Allison, Ezra Aydin, Simon Baron-Cohen, Rosemary Holt

**Affiliations:** University of Cambridge, UK

**Keywords:** autism, healthcare, maternity, motherhood, parenting, pregnancy, sensory processing

## Abstract

Pregnancy may pose a number of physical and healthcare-related challenges for autistic people, who experience differences in sensory processing and can face barriers to accessing healthcare. However, little research has explored autistic pregnancy experiences. Semi-structured interviews were conducted with 24 autistic and 21 non-autistic women during the third trimester of pregnancy. Thematic analysis revealed that the autistic group experienced heightened sensory and physical symptoms during pregnancy compared with the non-autistic group. Autistic participants were sometimes reluctant to disclose their diagnosis to healthcare professionals and felt that professionals lacked autism knowledge. While both groups appreciated clear information about their care, autistic participants further highlighted the need for detailed information and being given time to process verbal information. The autistic group also highlighted the need for sensory adjustments in healthcare settings. The findings indicate ways in which prenatal healthcare can be improved for autistic people, including sensory and communication adjustments. There is a need for greater autism-related training for prenatal healthcare professionals to ensure that autistic people receive appropriate support.

**Lay abstract:**

Little is known about how autistic people experience pregnancy. We interviewed 24 autistic and 21 non-autistic women during pregnancy to find out about their experiences. Autistic participants had more physical difficulties, such as nausea and pain, during pregnancy than non-autistic participants. They also sometimes felt that healthcare professionals, such as midwives, did not have a good understanding of autism and they did not always feel comfortable telling professionals about their autism diagnosis. Autistic participants told us that they needed professionals to communicate with them clearly and to make changes during appointments such as dimming lights. This research shows that autistic people would benefit from changes to pregnancy appointments and that more training about autism would help maternity care professionals to support autistic people during pregnancy.

## Background

Autism is a neurodevelopmental condition, characterised by differences in social interaction and communication, restricted and repetitive behaviours and sensory processing differences (American Psychiatric Association, 2013). Pregnancy can be physically demanding, with issues such as nausea (Heitmann et al., 2017), sensory changes (Cameron, 2014) and pelvic pain (Gutke et al., 2017) affecting quality of life. Good communication and respectful relationships with healthcare providers are important to ensure appropriate maternity care (Rowe et al., 2002). Sensory and communication differences may therefore create additional challenges during pregnancy for autistic people. However, research exploring autistic experiences of pregnancy is scarce.

Research into the parenthood experiences of women with disabilities more broadly indicates several challenges. Women with intellectual disability (ID), for example, can feel uninvolved in choices about their maternity care (Malouf et al., 2017) and may benefit from an advocate to support them with communication (McGarry et al., 2016). Mothers with ID and mothers with mental health conditions are also more likely to encounter social services (Booth & Booth, 2005; Park et al., 2006) and can be reluctant to disclose difficulties to professionals for fear of judgement (Malouf et al., 2017; Montgomery et al., 2006). Mothers with mental health conditions also report having limited social support (Montgomery et al., 2006) and can benefit from peer support to combat isolation (Alakus et al., 2007; Diaz-Caneja & Johnson, 2004).

Furthermore, maternity care professionals can feel they lack sufficient training to provide care for women with ID (Castell & Stenfert Kroese, 2016; Homeyard et al., 2016), women with physical conditions (Smeltzer et al., 2018) and women with mental health conditions (Higgins et al., 2016). Research into the perspectives of maternity care professionals when caring for autistic patients is scarce, although other healthcare professionals report that they lack training about autism in adults (Morris et al., 2019; Urbanowicz et al., 2020; Zerbo et al., 2015). Furthermore, autistic adults can feel that professionals’ lack of autism knowledge can prevent them from receiving appropriate care (Nicolaidis et al., 2015). Autistic women in particular report that their ability to hide their autistic characteristics can lead to healthcare providers underestimating their needs (Tint & Weiss, 2018). Autistic people can also face communication barriers to healthcare, including challenges processing verbal information during appointments (Raymaker et al., 2017) and a lack of non-verbal information resources (Nicolaidis et al., 2015). Differences in sensory processing can also make the sensory environment of healthcare facilities challenging (Raymaker et al., 2017).

The limited existing literature on pregnancy experiences of autistic people echoes the barriers identified above. Rogers et al. (2017) conducted a case study of an Australian autistic woman who reported heightened sensory experiences during pregnancy. The woman felt that healthcare professionals had little understanding of autism and that they did not respect her wishes nor treat her respectfully. Another qualitative study involving eight autistic women who commented on their experiences retrospectively, revealed enhanced sensory sensitivities during pregnancy which made aspects of prenatal appointments, such as bright lights and touch, challenging (Gardner et al., 2016). The mothers were sometimes reluctant to disclose their diagnosis to professionals and emphasised a preference for clear and direct communication. One quantitative survey study found that autistic mothers often chose not to disclose their diagnosis to professionals (e.g. teachers, clinicians, social workers) due to concern that their attitude towards them would change (Pohl et al., 2020). Autistic mothers were also more likely than non-autistic mothers to report difficulty communicating with professionals about their child, and greater difficulty with aspects of parenting such as multi-tasking.

In addition to increased challenges with physical aspects of pregnancy, such as sensory changes, and increased barriers to accessing adequate healthcare, autistic people may also experience poorer mental health during pregnancy. The fact that autism and mental health conditions often co-occur (Lai et al., 2019), and that a prior history of mental health conditions is a risk factor for worse perinatal mental health (Lancaster et al., 2010), may dispose autistic people towards lower mental well-being during pregnancy. Furthermore, a lack of social support during pregnancy has been associated with lower well-being (Elsenbruch et al., 2007). Given associations between autism and increased loneliness (Ee et al., 2019), autistic people may be more vulnerable to feelings of isolation during pregnancy, which may in turn affect well-being during this time. Indeed, one study found that autistic mothers were more likely than non-autistic mothers to report having had prenatal and postnatal depression (Pohl et al., 2020).

In sum, autistic people may face increased challenges during pregnancy, including sensory and communication challenges that may impact their experience of pregnancy-related care. However, research focusing on autistic pregnancy experiences using larger samples and non-retrospective methods (which may yield greater accuracy of reporting than retrospective methods) within a predominantly UK context is lacking. For this study, predominantly UK-based autistic women and a comparison group of non-autistic women were interviewed once during the third trimester of pregnancy, to explore pregnancy experiences.

## Method

### Participants

Participants were 24 autistic women and 21 non-autistic women. A diagnosis of autism was established based on self-report. Twelve autistic participants and all non-autistic participants participated as part of a larger study exploring the development of children with an autistic mother or sibling (the Cambridge Human Imaging and Longitudinal Development (CHILD) study). In addition to those participants involved in the present study, the CHILD study cohort also included three non-autistic mothers with an autistic child who are not reported on here. The remaining participants in the present study participated as part of another study exploring autistic mothers’ well-being (the Perinatal Experiences and Autism study), for which participants completed the semi-structured interviews reported here in addition to questionnaires concerning well-being (reported elsewhere; Hampton, Allison, et al., 2022). Participants were recruited through the ultrasound unit of the Rosie Maternity Hospital in Cambridge, the Cambridge Autism Research Database (CARD), autism-related and pregnancy-related support groups, social media and magazine advertisements. Those younger than 18 years were excluded. Ethics approval for the Perinatal Experiences and Autism study was obtained from the University of Cambridge Psychology Research Ethics Committee (PRE.2018.050). The CHILD study received NHS ethics approval (REC reference number: 12/EE/0393). Interviews concerning postnatal experiences were also conducted with the same participants 2–3 months after giving birth, and these data are reported elsewhere (Hampton, Man, et al., 2022).

All infants were born at 36 weeks gestation or greater. All participants identified as women. All participants were in a partnership apart from one autistic participant. The autistic groups were significantly younger, less likely to be non-white, had a lower level of education and lower household income, were more likely to have previously been diagnosed with a co-occurring psychiatric condition and had higher Autism-Spectrum Quotient (AQ) scores than the non-autistic mothers (Table 1). The groups did not significantly differ in countries, number of children, pregnancy conditions or age of the child at the time of interview.

**Table 1.** Demographic information for the autistic and non-autistic groups.

	Autistic (*n* = 24)	Non-autistic (*n* = 21)	*p*-value
Mean age (*SD*)^a^	31.10 (4.14) *range* *=* *21.56–35.76*	33.30 (2.44) *range* *=* *27.88–37.47*	**0.03**
Mean age of child in gestational weeks (*SD*)^a^	32.29 (2.60) *range* *=* *30.00–39.57*	31.01 (2.39) *range* *=* *29.71–34.00*	0.09
Ethnicity^b^			**0.04**
White	24 (100%)	16 (76%)	
Non-white	0 (0%)	5 (24%)	
Educational level^b^			**0.02**
Undergraduate or above	14 (58%)	19 (90%)	
A level or below^c^	10 (42%)	2 (10%)	
Annual household income (£)^b^			**0.001**
>50,000	7 (29%)	18 (86%)	
⩽50,000	17 (71%)	3 (14%)	
Psychiatric conditions^b^			**0.003**
None	8 (33%)	19 (90%)	
Depression	2 (8%)	1 (5%)	
Depression and anxiety	7 (29%)	1 (5%)	
OCD and anxiety	2 (8%)	0 (0%)	
Other	5 (21%)	0 (0%)	
Country of residence^b^			0.06
UK	19 (79%)	21 (100%)	
USA	4 (17%)	0 (0%)	
Ireland	1 (4%)	0 (0%)	
Number of children (not including current pregnancy)^b^			0.10
0	18 (75%)	12 (57%)	
1	2 (8%)	7 (33%)	
2	4 (17%)	2 (10%)	
Pregnancy conditions^b^			0.32
Gestational diabetes	5 (21%)	1 (5%)	
Polyhydramnios	1 (4%)	0 (0%)	
Pre-eclampsia	0 (0%)	1 (5%)	
Mean AQ score (*SD*)^a^	39.8 (5.54) *range* *=* *24–45*	15.2 (7.67) *range* *=* *6–30*	**<0.001**

*SD*: standard deviation.

a *T*-test performed.

b Fisher’s exact test performed.

c A-level approximately corresponds to the 12th grade in the US education system.

Bold values indicates the significance for *p* < .05.

### Measures

#### Semi-structured interviews

Semi-structured interviews, lasting 20–60 min, were conducted during the third trimester of pregnancy. All participants gave written or electronic informed consent. Interviews took place in person or remotely (*via* video call or telephone) between 2017 and 2019. A script of open-ended questions guided the interviews (available in the supplementary material). Topics included the physical and sensory experiences of pregnancy as well as interactions with healthcare professionals. The topics included were chosen based on prior literature and the input of an autistic mother (see the Community involvement section below).

#### The Autism-Spectrum Quotient (AQ)

Participants completed the AQ (Baron-Cohen et al., 2001), a self-report measure of autistic traits. Scores range from 0 to 50, with higher scores indicating greater autistic traits and a score of 32 or above indicating potentially clinically significant levels of autistic traits.

### Data analysis

The Research Electronic Data Capture platform (Harris et al., 2019; Harris et al., 2009) was used to record demographic data. Interviews were audio recorded and transcribed by the first author. Interviews were analysed (using NVivo software; version 12) according to a process of inductive, thematic analysis as outlined by Braun and Clarke (2006). This method focuses on extracting themes from the data without relying on pre-existing theories, making it appropriate for under-researched topics.

**Figure 1.**
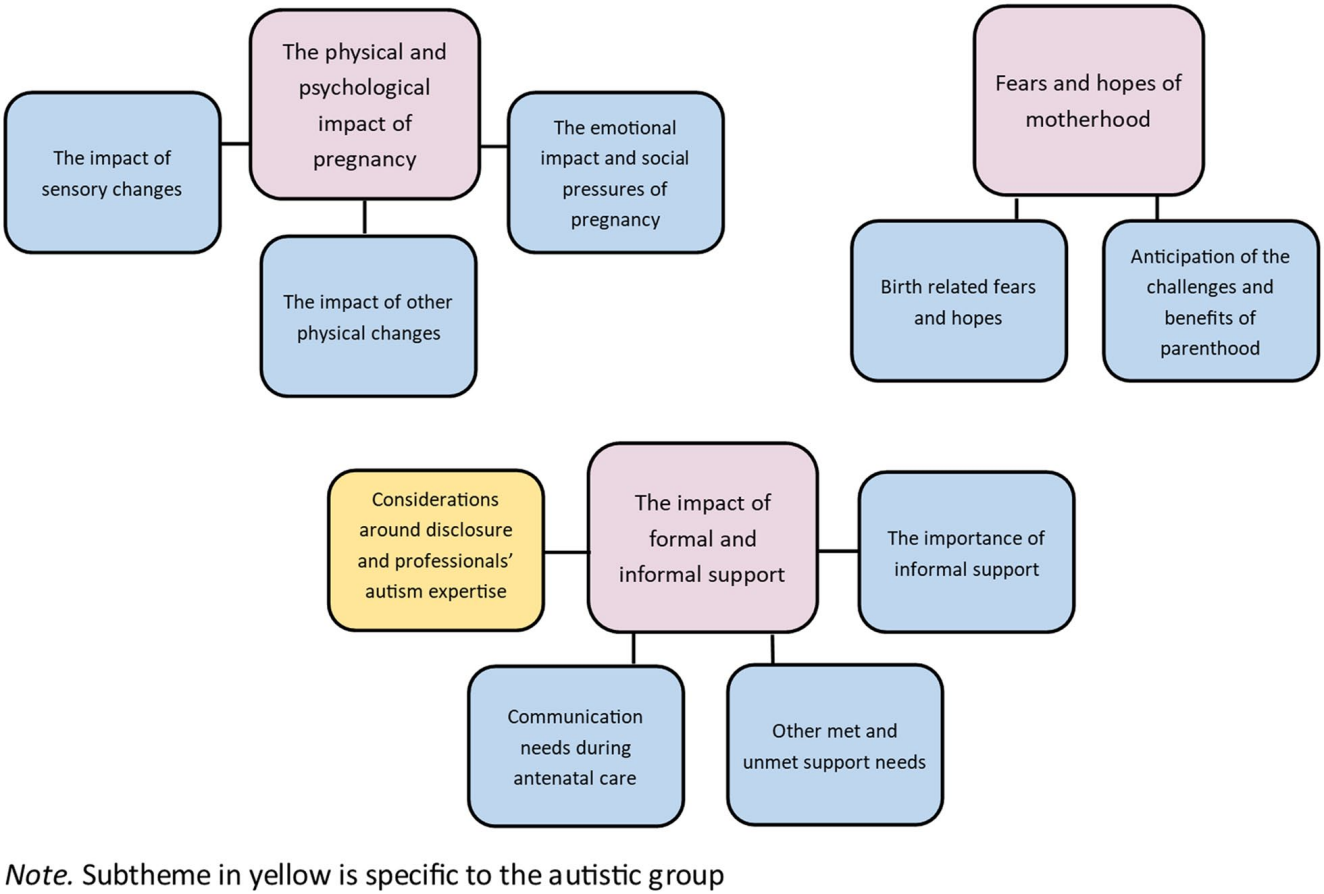
Themes and subthemes for the autistic and non-autistic groups.

Following data familiarisation, each interview was analysed line-by-line for initial codes. Next, initial codes were grouped into midlevel subthemes and final-level themes. Themes and subthemes were checked for internal coherence and lack of overlap by removing, splitting or combining them where necessary. Data from the autistic and non-autistic groups were analysed together. That is, initial codes, midlevel subthemes and final-level themes were generated for the full dataset, rather than for each group separately. Some subthemes arose only for the autistic group (Figure 1) and while other themes and subthemes arose for both groups, group differences in the manner in which these themes and subthemes were expressed often arose. These group differences in expression are brought out in the results section below. A consensus approach (Barker & Pistrang, 2005) was used in which the first author took the lead in the analysis and themes were revised with the second author during regular discussions at each stage of the analysis. 10% of the transcripts (split evenly across the autistic and non-autistic groups) were coded by the second author according to the themes and subthemes already generated and Cohen’s kappa (Cohen, 1960) was calculated as a measure of inter-rater reliability. Kappa values of 0.00–0.20 are considered slight, 0.21–0.40 fair, 0.41–0.60 moderate, 0.61–0.80 substantial and 0.81–1.00 as near-perfect agreement (Cohen, 1960). If Cohen’s kappa was below 0.70 for any theme or subtheme, this theme or subtheme was discussed and revised and 10% of transcripts were again coded by the second author. The initial mean kappa of all themes/subthemes was 0.78 (range = 0.53–1.00). The final mean kappa was 0.91 (range = 0.78–1.00).

The authors approached the research from the perspective of autism researchers who did not themselves have a diagnosis of autism. They approached the study with the intention of producing research that would be of benefit to the autistic community. All but the third and fifth authors did not have personal experience of parenthood. The research team were all educated to the post-graduate level, and all but the fifth author identified as women.

### Community involvement

The interview script was developed in consultation with an autistic mother to ensure that the content reflected relevant issues and that the wording was acceptable to the autistic community. Feedback on a draft of the manuscript was given by another autistic mother to help ensure the interpretation of results was acceptable to the autistic community.

## Results

Results are presented for both groups together for ease of comparison. Pseudonyms are used throughout to preserve anonymity.

Three themes, comprising nine subthemes, were identified (Figure 1) (1) ‘The physical and psychological impact of pregnancy’; (2) ‘The impact of formal and informal support’; and (3) ‘Fears and hopes of motherhood’.

### The physical and psychological impact of pregnancy

This theme explored the impact of the bodily, emotional and social changes that accompany pregnancy. Three subthemes emerged: (1) ‘The impact of sensory changes’; (2) ‘The impact of other physical changes’; and (3) ‘The emotional impact and social pressures of pregnancy’.

#### The impact of sensory changes

Participants in both groups commonly reported increased sensitivity to smells and tastes during pregnancy, ‘my smell is much, much more sensitive than it was and certain smells now I really dislike’ (Leah, non-autistic). While sensory changes in the non-autistic group were limited to smell and taste, the autistic group commonly reported changes concerning sound, lights, and touch, ‘I have like a sensory processing disorder with noises and light touching and smells and sounds and all of that is magnified and amplified’ (Sally, autistic). One participant reported heightened synaesthesia, ‘if I hear a really loud car horn or something for example, it feels like I’m being hit by something. So that one has got a lot more intense since being pregnant’ (Juliette, autistic).

The autistic group often found these sensory changes overwhelming and this could make coping with day-to-day tasks more challenging, ‘some things that I would be able to cope with normally, I wouldn’t be able to cope with or would stress me out even more. Just general things like the supermarket and stuff’ (Isla, autistic). Some autistic participants linked sensory issues to an increase in meltdowns and shutdowns.

it just kind of comes on very suddenly and a lot more intensely than before, so that’s where the coping strategies that I had before don’t really work. (Juliette, autistic)

#### The impact of other physical changes

This subtheme explored morning sickness, pain, tiredness and adapting to changes in body size. Some participants in both groups reported few issues with morning sickness, while others encountered greater challenges. While non-autistic participants with morning sickness tended to report it abated after the first trimester, ‘it waned at around the sort of time that it was supposed to wane, so that was all right’ (Vanessa, non-autistic), some autistic participants experienced sickness throughout the entire pregnancy. Three autistic participants reported that morning sickness disrupted their work and several autistic participants reported experiencing very frequent vomiting, ‘at one point I was vomiting 20 times a day’ (Beatrice, autistic). Two autistic participants felt that this was linked to sensory issues, ‘maybe it is worse for people with a sensory aversion to smell anyway because it’s heightened’ (Isla, autistic).

Several members of the autistic group talked about joint and ligament pain, with three autistic participants experiencing pelvic girdle pain. Two of these participants linked their pain to hypermobility, ‘I know ASD can be associated with loose ligament issues, but my hips for three out of the four [children] completely unravelled’ (Clarissa, autistic). One non-autistic participant described similar issues with pelvic pain.

While both groups talked of physical fatigue, several members of the autistic group additionally commented on mental fatigue and its impact on information processing abilities.

things that autism generally makes harder for me, so if I need to go into a store and process lots of different options, I don’t have the energy to do that anymore. (Simone, autistic)

Another participant commented, ‘Because you’re more tired and you’re thinking about a lot of things when you’re pregnant, I find speech has been a lot more difficult to understand or process’ (Juliette, autistic). The autistic group additionally found it challenging to adjust to rapid changes in body size and shape.

with my body changing shape, my centre of gravity changing, my balance changing, it feels like, OK, I’ve had 30 years to get used to this body and now it’s different, the rules have changed. I have to figure out new ways of moving and being in my body. (Simone, autistic)

#### The emotional impact and social pressures of pregnancy

This subtheme relates to positive and negative emotions during pregnancy and feelings surrounding the social attention that pregnancy attracts. Participants in both groups discussed positive emotions such as enjoyment and excitement, though some participants in both groups felt that negative emotions were heightened, ‘emotions are amplified as well and I start to cry for nothing’ (Diana, non-autistic). Participants in the autistic group also mentioned increased anxiety and low mood, with some linking these changes to hormonal influences, ‘I find that I’m pretty hormonally sensitive, which talking to other women with Asperger’s I think they are too. So I’ve just been really moody and extra anxiety’ (Olivia, autistic).

Both groups mentioned that being pregnant attracted social attention. The non-autistic group tended to find these conversations pleasant, though some found them boring or did not enjoy being the centre of attention. The increase in social attention was sometimes experienced as tiring by the autistic group, ‘they just come up to you and say, ‘Oh how long left?’, or ‘How many months are you?’, ‘Is it a boy or a girl?’, and in everyday life that’s exhausting for me’ (Lily, autistic). Some autistic participants felt pressure to respond in a normative way, ‘I’m supposed to act a certain way, give certain answers when people ask me, ‘Isn’t being pregnant great?’ (Olivia, autistic). However, some members of the autistic group appreciated that conversations about their pregnancy gave them a social script, ‘there’s a thing to talk about, there’s a baby coming so people say stock things to you and you say stock things back’ (Beatrice, autistic).

### The impact of formal and informal support

This theme explored experiences with both professional and informal support. Four subthemes emerged: (1) ‘Considerations around disclosure and professionals’ autism expertise’; (2) ‘Communication needs during antenatal care’; (3) ‘Other met and unmet support needs’; and (4) ‘The importance of informal support’.

#### Considerations around disclosure and professionals’ autism expertise

This subtheme explored considerations around disclosure of an autism diagnosis, professionals’ reactions to disclosure and professionals’ knowledge of autism. Participants who disclosed their diagnosis to professionals did so to bring about improvements in care, while those who did not worried that professionals would react negatively, ‘some medical professionals think that Asperger’s is a kind of hypochondriac fake excuse disorder so I’m afraid that if I brought up other concerns maybe they would treat me differently’ (Olivia, autistic).

Participants sometimes felt that disclosure was met with disbelief due to professionals lacking knowledge of autism among women, ‘I had a doctor the other day say, ‘I’ve worked with autistic kids, and you’re not like them’. And I was like, ‘OK, I’m probably not, and probably they’re mainly boys as well’ (Debbie, autistic). Participants sometimes felt that professionals’ lack of autism knowledge was a barrier to having their needs met, ‘I mentioned it at the first appointment and she was a bit like, ‘oh, what does that mean?’ and I had to explain it. But she’s not really brought it up since then’. However, some felt that lack of awareness could be compensated for by an individualised approach, ‘[My midwife] doesn’t have a lot of experience of autism but she listens to what I have to say about my experiences and then she adapts’ (Juliette, autistic).

Participants sometimes felt that professionals’ lack of autism awareness led to a break down in trust. One participant reported that discussing meltdowns with her midwife led to an unwarranted referral to social services.

I feel like if I say that I’m struggling they’re going to forget all the ways in which I’m coping well. And like with the social services thing being triggered, it has made me feel a bit not sure about what I can and can’t say without it being misunderstood. (Morgan, autistic)

Others echoed this lack of understanding,

I’ve been asked by a couple of the midwives how I think I can be a mum if I’m autistic. [. . .] I would never put my daughter in danger, but there’s been very much a feeling that that would be a possibility. (Debbie, autistic)

#### Communication needs during antenatal care

Participants in both groups emphasised the need for clear information surrounding their care, ‘they just haven’t mentioned something I’ve come across on the Internet or friends have told me about and I find it a little bit disconcerting’ (Cassandra, non-autistic). The autistic group emphasised a need for detailed, factual information, including clear information about what to expect in appointments.

If everything could be structured and written down so that I could see, ‘this week you’re going to see this person, these are the things we’re going to talk about, these are the possible outcomes’. (Beatrice, autistic)

Others echoed this need for written information, ‘she writes things down as she’s saying them and then gives me the notes so that during the appointment if I’ve kind of lost myself halfway through I can always read the note afterwards’ (Juliette, autistic). Autistic participants also appreciated extra time in appointments to process verbal information and ask questions. Processing information over the phone could be challenging and some preferred email, text or in-person communication, *‘*I have a lack of phone contact and face to face only, so [my partner] does every email and phone contact for me and that has made all sorts of awkwardness. Access to services is really hard’ (Debbie, autistic). The autistic group also reported that specific rather than open-ended questions helped elicit accurate responses,

if someone says, ‘How are you?’, I just say, ‘Alright’. Whereas if someone said to me, ‘How’s your pelvic pain?’, I’d say, ‘It’s been terrible’, or ‘It’s okay’. If it was more specific, I’d probably answer it a bit more. (Beatrice, autistic)

Some autistic participants felt that having an advocate was helpful for communication.

I’ve always tried if I can to have my mum with me at the appointments, because I do struggle sometimes to take in things they say to me, and because I struggle to take things in I do then end up getting railroaded into making decisions that I might not actually agree with. (Jolene, autistic)

Autistic participants sometimes felt that professionals dismissed their knowledge of their bodily experiences.

I may not be good at reading people, but I’m really good at reading my body. And I’m not a hypochondriac, I can say something is happening and it’s happening, which I guess people think is kind of weird, they don’t believe you. (Olivia, autistic)

Another autistic participant commented, ‘she sort of said, ‘oh pregnancy will be uncomfortable’ and I wasn’t sure whether it was me being pathetic and hyper-sensitive to pain or whether it was her not realising how much pain I was in’ (Tara, autistic).

#### Other met and unmet support needs

Participants discussed their experiences with continuity of care, sensory issues and antenatal classes. Continuity of care was often desirable but non-essential for non-autistic mothers, ‘I never had the same midwife for any of the appointments I’ve had. Because they don’t know you it feels like you might get less support, but at the same time, I’ve not had any issues’ (Sadie, non-autistic). The autistic group valued continuity of care for building trust and understanding, ‘she understands me so it’s helpful having her instead of having to explain or having someone else who doesn’t understand’ (Ethyl, autistic), as well as for ease of communicating their medical history.

During appointments, some members of the autistic group found the sensory environment of the hospital challenging, ‘I find it really hard in the waiting room where I see the midwives because they often have music on and the lights are really bright and it’s just horrible’ (Pearl, autistic). For one participant, a negative hospital experience had an enduring impact, ‘it was so chaotic and bright and people rushing around and not very direct advice. I found that really difficult and I shutdown for a period afterwards, after my first scan’ (Yvette, autistic).

Some participants in both groups valued antenatal classes as a way of meeting other parents, ‘it is nice going through similar things with parents that are in the same sort of bracket as you’ (Lisa, non-autistic). Members of the autistic group, however, sometimes found the social aspect challenging and some found smaller classes, online classes, or one-to-one classes with a midwife or doula preferable, ‘I hired a doula who’s coming to my home to do it and that’s better for us because I don’t like big crowds and groups’ (Sally, autistic).

#### The importance of informal support

This subtheme explored support from partners, family and friends. Both groups tended to feel well supported by their partners and family and the non-autistic group valued the support of friends who were also parents, ‘it’s been really helpful to share experiences’ (Rozetta, non-autistic). The autistic group sometimes identified a lack of support from friends, ‘And also having a lack of friends, I feel like I’ve got a lack of female . . . people who’ve been through pregnancy’ (Morgan, autistic). Autistic participants often felt they would benefit from peer support from other autistic parents, either through social media or in-person groups, ‘I don’t really know anyone else with autism who’s had a baby and there’s not really much out there to find out about it, so that’s been quite isolating as well’ (Irene, autistic).

### Fears and hopes of motherhood

This theme explored participants’ feelings as they look ahead to childbirth and beyond. Two subthemes emerged: (1) ‘Birth-related fears and hopes’ and (2) ‘Anticipation of the challenges and benefits of parenthood’.

#### Birth-related fears and hopes

Childbirth often represented an unknown for both groups, particularly for those who had not previously given birth, ‘it is frightening to think about certain elements of delivery or if you imagine things one way but then if it doesn’t go a certain way’ (Lisa, non-autistic). With the autistic group, worries around uncertainty were often linked to a desire for predictability, ‘there’s the uncertainty of when it’s going to be and how long it’s going to take and what’s going to happen, that uncertainty is adding to my fear of it’ (Melinda, autistic). Some autistic participants felt that a detailed birth plan and visiting the labour ward in advance would help with these concerns,

just like the room I’m going to be in or the ward, that sort of thing would make a huge difference to me, just so I can anticipate what it sounds like, what it smells like, that would really help. (Pearl, autistic)

The autistic group expressed concerns about communication with professionals, ‘am I going to be able to not just communicate during labour but to understand what people are saying to me? If I’m being given any instructions to push or whatever, how am I going to process that?’ (Juliette, autistic). Autistic participants also expressed concerns that professionals would not keep them adequately informed, ‘they’ll downplay things, whereas for me I would much rather be told what’s going on, what the consequences of that are, what that means’ (Isla, autistic). While communication concerns were expressed by both primiparous and multiparous participants, these worries were particularly common among those who had not previously given birth.

Autistic participants also commented on the potential sensory challenges of hospitals,

I’m due to go into one of the birthing centres where you can have your own music on and the lights are quite low and they don’t have a lot of people coming in and out. I think that will help me a lot with my sensory issues. (Jolene, autistic)

Some chose a home birth to avoid the challenges of the hospital environment, ‘At home you have control over your environment, you can control the lighting, the noise, you know exactly who is going to be in the room’ (Clarissa, autistic).

#### Anticipation of the challenges and benefits of parenthood

Both groups looked forward to motherhood with excitement, ‘I really, really can’t express how excited I am about being able to meet her and cuddle her’ (Paige, autistic) and looked forward to seeing their baby develop and learn, ‘you get to see them develop, you get to see them learn and see what’s exciting’ (Leah, non-autistic). Participants in both groups, particularly those who were first-time parents, expressed concerns about rising to the responsibility of motherhood, ‘It’s a big things babies, very important, and I want to make sure I do right’ (Yvette, autistic). Some autistic participants discussed wanting to be an understanding parent,

I don’t want to be one of these parents that’s like always just saying that their child’s naughty, I want to understand what’s triggering it rather than blaming and punishing, like try and understand. I don’t know whether that’s because I’ve felt misunderstood so much. (Morgan, autistic)

Some autistic participants worried about feeling isolated after birth,

I worry about being isolated. I know everyone says about going to mother and baby groups, which I will go to, but I just don’t want to sit in a room with a load of women I have nothing in common with. (Kayleigh, autistic)

Some autistic participants were concerned about the executive functioning demands of parenthood.

once I’ve got going I’m alright, but getting going can be very slow. I worry about looking after myself, cooking and things can be very difficult, so I guess support with that would be useful. (Yvette, autistic).

## Discussion

This study provides insights into the pregnancy experiences of autistic people and identifies areas where they can be better supported. The physical symptoms of pregnancy tended to be heightened for the autistic group, including experiencing sensory changes, morning sickness, joint pain, and mental fatigue more acutely. Increased sensory challenges echo prior reports of heightened sensory experiences during pregnancy (Gardner et al., 2016; Rogers et al., 2017) and fit with the presence of sensory sensitivities among autistic people more generally (Tavassoli et al., 2014). Participants speculated on these experiences including increased morning sickness due to sensory sensitivities and increased joint pain due to hypermobility. Healthcare professionals should be aware that autistic patients may experience heightened physical challenges; such awareness could allow for more effective identification and treatment of physical issues during pregnancy.

Some participants did not disclose their autism diagnosis to professionals for fear of negative reactions, similar to reports by Gardner et al. (2016) and Pohl et al. (2020). When participants did disclose, they often felt that professionals had limited awareness of autism among women and that adjustments were not made. These findings align with research showing that autistic women feel maternity care professionals lack autism knowledge (Rogers et al., 2017), as well as findings that maternity care professionals feel they lack sufficient training concerning ID and mental health (Castell & Stenfert Kroese, 2016; Higgins et al., 2016).

A lack of autism understanding among professionals occasionally led to participants feeling unable to reveal difficulties for fear of being misunderstood. Indeed, one autistic participant experienced a referral to social services that they felt was based on misunderstandings. This is consistent with findings that mothers with ID and mental health conditions are more likely to encounter social services (Booth & Booth, 2005; Park et al., 2006) and that they can fear being honest with professionals (Malouf et al., 2017; Montgomery et al., 2006). Greater autism-related training for maternity care professionals would allow greater understanding and trust between professionals and autistic people. Continuity of care was also identified by some autistic participants as important for building a sense of trust and may be an important adjustment to make for autistic people. Given that mothers with conditions other than autism, such as mental health conditions and ID, can also feel a lack of trust and a reluctance to disclose difficulties to professionals (Malouf et al., 2017; Montgomery et al., 2006), adaptations that may support autistic people to disclose their diagnosis might also be beneficial in supporting people with other neurodevelopmental and mental health conditions to disclose to maternity care professionals.

The autistic group emphasised the importance of receiving clear, factual information from professionals, including what to expect in appointments and who they would see. Autistic participants sometimes experienced difficulties processing verbal information in appointments and preferred written information and alternatives to phone communication. These preferences are in keeping with findings of communication barriers to healthcare for autistic people (Nicolaidis et al., 2015; Raymaker et al., 2017) and reports that autistic women require clear and direct information when interacting with maternity care professionals (Gardner et al., 2016). The findings therefore indicate the need to make communication adjustments for autistic people, including the provision of clear information, as well as information in a range of accessible formats. In line with research suggesting that an advocate can be beneficial for mothers with ID (McGarry et al., 2016), some autistic participants found the presence of an advocate helpful for communication during appointments. Autistic participants sometimes found the social and communication demands of antenatal classes challenging. The provision of one-to-one classes or smaller classes may therefore be beneficial. In addition, autistic participants occasionally found the sensory environment challenging during prenatal appointments, echoing prior findings (Gardner et al., 2016) and highlighting the need for sensory accommodations.

When anticipating childbirth, autistic participants expressed concerns about communicating with professionals and coping with the sensory environment of the hospital. This shows the need for communication adjustments for autistic patients during childbirth as well as sensory adjustments such as dimming lights and minimising the number of professionals in the room. Anxieties surrounding childbirth were particularly common among first-time parents. Professionals should therefore be mindful of addressing birth-related anxieties among this group.

Both groups reported excitement about meeting their baby and watching them develop. The autistic group, however, expressed concerns about coping with the executive function demands of parenthood, indicating that autistic parents may benefit from support in this area. Furthermore, autistic participants expressed concerns about feeling isolated after birth and sometimes felt they lacked support from friends. Consistent with findings establishing the value of peer support among mothers with mental health conditions (Diaz-Caneja & Johnson, 2004), the autistic group tended to feel that peer support from other autistic parents was desirable. Peer support for autistic parents may therefore help reduce the risk of isolation.

### Limitations

The non-autistic group all resided in the United Kingdom and as such, their healthcare experiences may have been less varied than the autistic group who resided throughout the United Kingdom, the United States and Ireland. While differences in experiences across healthcare systems did not emerge as a theme from the data, it is nevertheless possible that some differences in healthcare experiences between autistic and non-autistic participants were influenced by this factor. The non-autistic group had a higher level of education and income than the autistic group. This may have afforded them access to better healthcare and as such some group differences in healthcare experiences may be amplified by socio-economic differences. The autistic group were more likely to have co-occurring mental health conditions. It is possible that some experiences of this group (such as feeling dismissed by professionals, contact with social services and anxiety surrounding group-based support) were influenced by the presence of other such conditions. The findings may therefore be less applicable to those autistic people who are not first-time parents, who have higher income and education or who do not have co-occurring psychiatric conditions. Furthermore, the researchers were not blind to the group membership of the participants and their interpretation of differences between the groups may have been influenced by any biases they hold.

The study could only capture the experiences of those with the verbal ability to take part in an interview and those who felt able to dedicate the time and energy to take part. Furthermore, parents experiencing challenging circumstances may have been unwilling to take part due to fear of disclosing difficulties. The study therefore likely only captures the experiences of a subset of the autistic community.

## Conclusion

The findings highlight a need for greater autism training for professionals involved in prenatal care. This, in addition to continuity of care, would help build trust and avoid misunderstandings between professionals and autistic patients. Professionals should be aware that autism can present differently between individuals, necessitating an individualised approach. The findings support providing accommodations surrounding communication during prenatal appointments, including provision of clear, factual information, written information and alternatives to telephone contact. The results indicate a need for sensory accommodations, including dimming lights, minimising noise and providing a quiet waiting area. The provision of one-to-one antenatal classes may also be preferable. Despite group differences, the findings also highlight commonalities in the experience of motherhood and many adaptations to care may serve to benefit both autistic and non-autistic mothers.

Future research should seek the perspectives of prenatal healthcare professionals to understand their autism-related knowledge, the level of autism-related training they receive and the barriers they may face to providing care for autistic people. Certain themes, including the impact of sensory changes during pregnancy as well as the role of social scripts around pregnancy for autistic women, could be given more in-depth exploration in future work. Future quantitative studies could establish the extent to which the themes identified generalise to a larger sample. Such studies could include surveys of autistic people’s pregnancy experiences as well as the use of health record data to explore physical pregnancy symptoms among autistic people.

## Supplemental Material

sj-docx-1-aut-10.1177_13623613221132435 – Supplemental material for A qualitative exploration of autistic mothers’ experiences I: Pregnancy experiences Supplemental material, sj-docx-1-aut-10.1177_13623613221132435 for A qualitative exploration of autistic mothers’ experiences I: Pregnancy experiences by Sarah Hampton, Joyce Man, Carrie Allison, Ezra Aydin, Simon Baron-Cohen and Rosemary Holt in Autism
