# Supplementary material for: A qualitative exploration of autistic mothers’ experiences I: Pregnancy experiences
Source: Autism. 2022 Nov 3;27(5):1271–82. doi: 10.1177/13623613221132435 (PMC10291382; doi:10.1177/13623613221132435)
Supplement: sj-docx-1-aut-10.1177_13623613221132435 – Supplemental material for A qualitative exploration of autistic mothers’ experiences I: Pregnancy experiences [file sj-docx-1-aut-10.1177_13623613221132435.docx]

**Interview questions**

**Overall experience of pregnancy:**

- Could you tell me a bit about what being pregnant is like for you?

**Sensory/physical experiences:**

- What have the physical aspects of pregnancy been like for you?
- Have you noticed any changes since becoming pregnant in your sensory experiences?

**Relationships with professionals:**

- How would you describe your relationships with healthcare professionals throughout pregnancy?
- [autistic group only] Have professionals been aware of your autism diagnosis?
- [autistic group only] Is there anything that you would like professionals to understand about autism in relation to prenatal appointments?
- Do you feel you have had all the information you need throughout your pregnancy?

**Feelings about childbirth/parenthood:**

- How are you feeling about giving birth?
- How are you feeling about becoming a mother?

**Support:**

- Do you feel you have all the support you would like?
- What support do you think would be helpful for pregnant [autistic] people?
